# Regulatory mechanism analysis of signal transduction genes during rapeseed (*Brassica napus* L.) germination under aluminum stress using WGCNA combination with QTL

**DOI:** 10.3389/fpls.2025.1546572

**Published:** 2025-01-31

**Authors:** Chenyang Li, Ruili Wang, Jiana Li, Qingyuan Zhou, Cui Cui

**Affiliations:** College of Agronomy and Biotechnology, Southwest University, Chongqing, China

**Keywords:** *Brassica napus* L., aluminum stress, RNA-seq, WGCNA, QTL

## Abstract

As soil becomes more acidic, aluminum toxicity has emerged as a key issue impacting seed germination and crop productivity in such environments. Therefore, it is urgent to investigate the mechanism of the influence of aluminum stress on germination. In this study, we focused on one of the major bioenergy crops—rapeseed. Seeds of aluminum-sensitive (S) and aluminum-resistant (R) lines screened from the recombinant inbred lines (RILs) population of rapeseed were treated with 80 µg·ml^-1^ AlCl_3_ (ST, RT). Purified water served as the control (SC, RC). On the 3rd, 5th, and 7th day after treatment, the root tissue was collected for transcriptome sequencing. Utilizing MapMan software, the genes showing differential expression in S and R lines were assigned to the aluminum stress signaling pathway, resulting in the identification of 1036 genes. By weighted gene co-expression network analysis (WGCNA), five co-expressed gene modules associated with aluminum stress were discovered. A total of 332 candidate genes were screened by combining the genes related to aluminum stress signal transduction pathways with the module hub genes. Among them, 26 key genes were located in quantitative trait loci (QTL) with confidence intervals for germination-related traits of rapeseed under aluminum stress, and primarily distributed in 11 QTL regions, such as *qRDW-A09-1*, *qRDW-A10-1* and *qRGV-A01-2*, they were associated with relative root length (RRL), relative root dry weight (RDW), relative germination vigor (RGV) and relative bud length (RBL). The roles included transcription regulation, stress protein production, redox processes, hormone signaling, cell wall alteration, and calcium-based signal transmission. Compared with the R line, the S line exhibited quicker and stronger activation of genes related to aluminum stress signal transduction, suggesting that the S line was more responsive to aluminum stress. This research offers an empirical basis for identifying aluminum-resistant rapeseed varieties and investigating the molecular regulation of aluminum tolerance during germination.

## 1 Introduction

Aluminum (Al) is among the most plentiful metallic elements in the Earth’s crust, ranking third place following oxygen and silicon (Panda et al., 2009). Non-ionized or elemental aluminum is harmless to plants, humans, and the environment; however, aluminum ions (Al^3+^) or free aluminum [Al(OH)^2+^] can be highly detrimental (Yan et al., 2018). When the pH of the soil is less than 5.0, the soluble Al content in the soil will increase, reducing the utilization of soil nutrients and crop yield. Aluminum contamination in soil can significantly limit China’s development of sustainability of agriculture (Ma and Furukawa, 2003). Rapeseed (*Brassica napus* L.) is a significant oilseed and energy crop for industry in China, predominantly cultivated in areas along the Yangtze River. However, much of the soil there is acidic, reducing the rapeseed yield because of Al stress (Kochian et al., 2004). Seed germination is the most important and vulnerable stage in the growth cycle of rapeseed (Gao et al., 2021). During this period, rapeseed is more sensitive to aluminum toxicity. Once the external Al^3+^ concentration is too high, the ability of rapeseed to expel aluminum is limited, and the internal regulatory system could not ensure the balance of various physiological systems, which would inhibit seed germination and radicle growth (Sami et al., 2020). Severe aluminum stress may also lead to the death of rapeseed plants, and then lead to the decline of grain yield and quality (Zhao et al., 2020). Therefore, studying the effect of aluminum toxicity on seed germination of rapeseed can avoid more negative factors brought by aluminum toxicity to the subsequent growth and development, and lay a good foundation for improving the yield and quality of rapeseed.

Research indicated that aluminum tolerance in plants is a complex trait influenced by several minor or major effectors, quantitative trait loci (QTL). Some QTL sites associated with Al tolerance had previously been identified in RILs (recombinant inbred lines) of wheat (Sara et al., 2020), rapeseed (Wang et al., 2020) and soybean (Li et al., 2021). But because of the large coverage area, a large number of candidate genes are usually obtained. Transcriptome sequencing (RNA-seq), with its high resolution and sensitivity, has been widely used to study the changes of plant gene expression under Al stress, such as tea plant (Huang et al., 2021), *Pinus massoniana* (Wang et al., 2022), and maize (Pinto et al., 2023). Correspondingly, RNA-seq results can also detect a large number of differentially expressed genes (DEGs) concerned with Al stress, but narrowing them down to key genes remains difficult. Therefore, combining transcriptome analysis with QTL genetic mapping can greatly reduce the number of candidate genes by selecting only those key genes related to crop resistance under abiotic stress, such as salt tolerance gene in rice (Geng et al., 2023), cold tolerance gene in chickpeas (Kalve et al., 2023), and heat tolerance gene in *Camelina sativa* (Smith et al., 2024). Weighted gene co-expression network analysis (WGCNA) is a crucial technique for investigating gene roles via network diagrams (Langfelder and Horvath, 2008), and it is instrumental in analyzing particular characteristics and identifying key genes from diverse plant transcriptome datasets. Ma et al. (2022) had identified a gene (*ZMHIPP*) associated with lead accumulation and tolerance in maize using a combination of linkage mapping and WGCNA, while Yin et al. (2024) used WGCNA and QTL mapping to screen the key genes *Os05g0498700* and *Os01g0866100* for anaerobic germination tolerance in rice. All of these studies demonstrated the effectiveness of combining WGCNA and QTL mapping to mine key genes for crop resistance. Nevertheless, the combination of WGCNA and QTL analysis to reveal the mechanism of aluminum resistance during rapeseed germination has not been documented.

The adaptability of plants to adversity depends on whether they can activate the molecular cascade network involved in stress signal recognition, signal transduction, and expression of stress-related genes and metabolites (Huang et al., 2012). Signal transduction processes can improve the adaptability of plants to adverse environmental conditions by forming highly ordered signal regulation networks (Zhu, 2016). In acidic soil, there are two kinds of plant adaptation mechanisms to Al stress: external Al exclusion and internal Al tolerance. The Al exclusion mechanism involves OAs (organic acids) secreted by plant roots for Al^3+^ chelation. Mechanisms of internal Al tolerance include the fixation of Al in the cell wall, the chelation of Al by OAs in the cytoplasm, or the separation of Al into the vacuole. Although different plant species have the same or similar regulatory mechanisms in response to Al, there are still subtle differences between them based on the signaling pathways activated by Al (Liu et al., 2022). This research employed QTL mapping, RNA sequencing, and WGCNA to explore the impact of aluminum stress on the changing transcriptome of the Al-sensitive rapeseed line 27011 and the Al-resistant line 18D300, aiming at identifying signal transduction genes associated with aluminum stress.

## 2 Materials and methods

### 2.1 Plant material and aluminum treatment

Among a rapeseed population of 182 RILs, *Brassica napus* 27011 was selected as the aluminum-sensitive (S) line while 18D300 as the aluminum-resistant (R) line (Wang et al., 2021a). To investigate the effect of Al^3+^ on germination, 20 seeds of the S and R lines were separately put in Petri dishes on three layers of filter paper that were moistened with 3 ml of 80 µg ml^-1^ AlCl_3_•6H_2_O in Distilled Water (DW) (Wang et al., 2021b). DW served as control. Next, the seeds were put in an artificial illumination incubator at 25°C/20°C (light/dark), 16 h light/8 h dark, and 85% relative humidity. On the 3rd, 5th, and 7th days of treatment, the root length was measured, and a 0.2 g root sample was collected into a 1.5 ml centrifuge tube, rapidly frozen in liquid nitrogen, and stored at −80°C for transcriptome sequencing and qRT-PCR verification. The control and treatment samples of the R line were named RC3, RC5, RC7, RT3, RT5, and RT7; likewise, the samples of S line were named SC3, SC5, SC7, ST3, ST5, and ST7, respectively. Each Petri dish was considered one biological replicate, with the R and S lines having five biological replicates at each control and treatment time point, resulting in a total of 30 biological replicates for both the R and S lines.

### 2.2 QTL mapping

Drawing from our earlier findings (Wang et al., 2020), a seed germination stress experiment was conducted on 182 samples of rapeseed. On the third day of treatment, the germination vigor was assessed. On the seventh day, ten seedlings with similar growth were randomly selected from each Petri dish for root length and bud length measurements. These ten seedlings were dried at 75°C for 24 h and their dry weight was measured. Using distilled water treatment for seed germination as a control, we calculated relative germination vigor (RGV), relative root length (RRL), relative bud length (RBL), and relative dry weight (RDW). We then conducted QTL mapping for RGV, RRL, RBL, and RDW in 182 lines of the RILs population, identifying candidate genes within the QTL confidence interval.

### 2.3 Transcriptome sequencing and DEGs analysis

Root samples from the S and R lines were dispatched to Personalbio Co., Ltd. (Shanghai, China) for RNA isolation, library preparation, and transcriptome sequencing using an Illumina platform. Following the removal of 3’-adapters and sequences with poor quality (sequence mass < Q20), the clean reads were aligned to the *B. napus* reference genome (http://www.genoscope.cns.fr/Brassicanapus/cgi-bin/gbrowse/colza/) utilizing HISAT2 software (http://ccb.jhu.edu/software/hisat2/index.shtml). The read count was determined by HTSeq (https://htseq.readthedocs.io/en/release_0.11.1/). Gene expression levels were estimated by calculating fragments per kilobase of exon model per million mapped reads (FPKM). To identify differentially expressed genes, DESeq (Wang et al., 2010) was employed, with screening criteria set at |log2(fold change)| ≥ 1 and p ≤ 0.05. The MapMan tool (Thimm et al., 2004) was employed to align the varying expressions of DEGs with the Al stress signaling pathway, identifying genes associated with Al stress response in both S and R lines.

### 2.4 Screening of key genes

A gene co-expression network was developed with the WGCNA package in R language (Langfelder and Horvath, 2008). The modules were generated with the automatic network construction feature (block-wise Modules) using default parameters, with slight adjustments (min Module Size = 30, merge Cut Height = 0.31). At each treatment time point for the S and R lines, distinct modules (module ME value |r| ≥ 0.70, trait correlation coefficient p ≤ 0.05) and hub genes (|characteristic gene connectivity| ≥ 0.8, |gene significance| ≥ 0.5) were identified. To minimize the count of crucial genes, hub genes identified through WGCNA were cross-referenced with differentially expressed genes (DEGs) filtered by MapMan software and subsequently aligned with the QTL physical regions associated with RGV, RRL, RBL, and RDW in rapeseed under Al stress (Wang et al., 2020). The corresponding sequences of key genes were queried in the *B. napus* genome, and then aligned with the *Arabidopsis* genome sequences on the TAIR website (https://www.arabidopsis.org/) by BLAST to determine the functions involved. KEGG metabolic pathway enrichment analysis was performed using the KOBAS2.0 platform (http://kobas.cbi.pku.edu.cn/home) as described by Xie et al. (2011). Pathways with FDR values below 0.01 were then identified as significant.

### 2.5 Quantitative RT-PCR validation

Quantitative real-time PCR tests were conducted with a kit provided by Beijing Labgic Technology Co., Ltd. (Beijing, China) using a BioRad CFX96 real-time cycler. Primer pairs were designed using qPrimerDB v1.2 (https://biodb.swu.edu.cn/qprimerdb/), produced by Sangon Biotech (Shanghai, China), and detailed in **Table 1**. The experimental parameters included an initial step of 95°C for 5 minutes, followed by 40 cycles consisting of 95°C for 10 seconds, 56°C for 30 seconds, and 72°C for 30 seconds. Using *BraActin7* as a reference for consistent expression, the gene’s relative expression was measured by the 2^-ΔΔCt^ methods (Zhao et al., 2019), with each sample having three biological replicates.

**Table 1:** List of primer sequences used for qRT-PCR analysis.

Gene ID	Forward primer (5’-3’)	Reverse primer (5’-3’)
*BraACTIN7*	GGAGCTGAGAGATTCCGTTG	GAACCACCACTGAGGACGAT
*BnaA03g54320D*	AACTACAAAGCAACAATGGTGG	GATCCTAAAGTCATCCGCAAAC
*BnaA01g28900D*	GTTGGCAATATTTCAGAAAGCG	TACAGTAGACTCCGTCTATCCC
*BnaA03g25630D*	GTGAAAATGCCGTTGAAGAGAT	GAAAAGGCAGAGTCTTTCTTGG
*BnaA08g02360D*	TCATCATAATCACCGTCACGAT	CCTGTTGGTAGTCAAAGAAACG
*BnaA03g52830D*	ATCGATGGTTGTTTAGGCTGTA	CCTCACTCCTCTGTATCCATTC
*BnaA01g27170D*	CGGTGTGTGTGTTTAACTTCAT	GATTAGAAAACGCCGGAATCAA

### 2.6 Data processing

Microsoft Excel 2019 was used to process the data and construct bar charts. Venn diagrams and gene expression heatmap of key genes were generated by TBtools software (Chen et al., 2020).

## 3 Results

### 3.1 Variations in the root length of S and R lines during various germination phases

**Figure 1:**
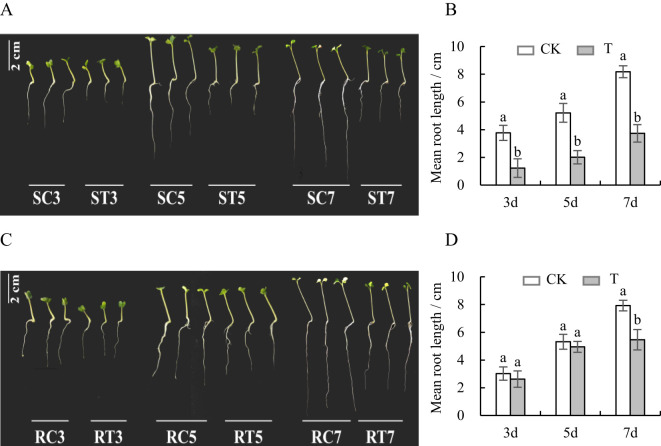
Variations in root length of S and R lines. **(A, B)** S line; **(C, D)** R line; CK is the control group, T is the Al-treatment group, and the different lowercase letters indicate significant differences at the p<0.05 level in each time period.

Compared with the control group, the root length of the S line was significantly shorter on the 3rd, 5th and 7th days after Al stress (*p* < 0.05) (**Figure 1A**), and a decrease rate was more than 50% (**Table 2**, **Supplementary Table S1**). The root extension of the R line was also inhibited, but the inhibition amplitude was less than 35%, which was much smaller than that of S line (**Figure 1B**; **Table 2**; **Supplementary Table S1**). This suggested notable variations in the tolerance of S and R lines to Al toxicity.

**Table 2:** Variance analysis of root length of S and R lines.

Time point	Treatment	S line	Comparison with the control (%)	R line	Comparison with the control (%)
3d	CK	3.77 ± 0.54a	-67.36	3.02 ± 0.48a	-13.18
	T	1.23 ± 0.67b		2.62 ± 0.59a	
5d	CK	5.21 ± 0.68a	-61.30	5.32 ± 0.54a	-6.85
	T	2.02 ± 0.48b		4.95 ± 0.39a	
7d	CK	8.19 ± 0.44a	-54.39	7.93 ± 0.38a	-31.02
	T	3.73 ± 0.63b		5.47 ± 0.74b	

Small letters in the table indicate significant difference between control and aluminum treatment (*p* < 0.05).

### 3.2 DEGs analysis

**Figure 2:**
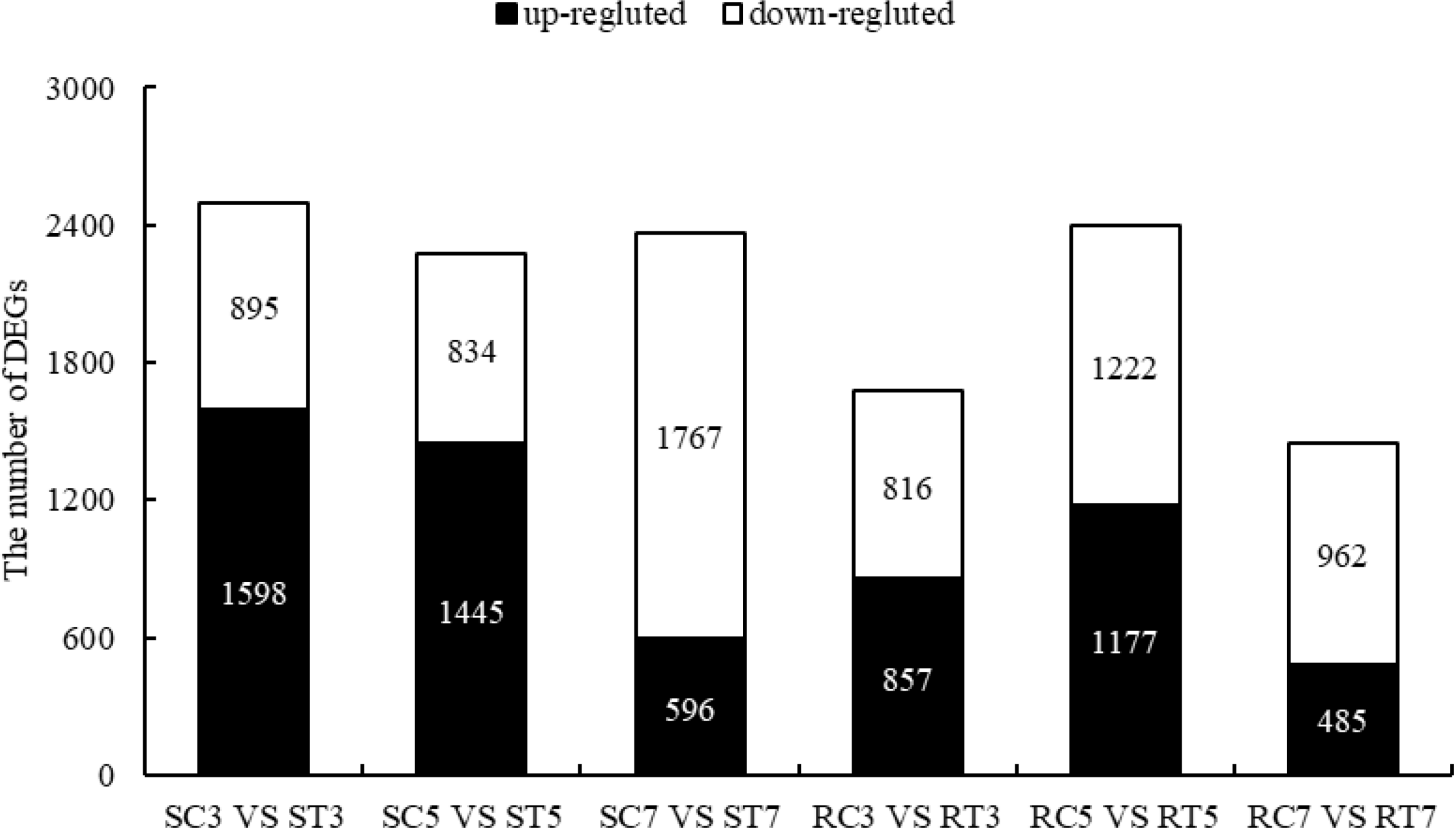
DEGs between S and R lines after aluminum treatment.

As shown in **Figure 2**, a total of 2493 and 1673 DEGs were obtained from SC3 *vs* ST3 and RC3 *vs* RT3, including 1598 (64.10%) and 857 (51.23%) up-regulated genes, 895 (35.90%) and 816 (48.77%) down-regulated genes, respectively. The 2279 and 2399 DEGs of SC5 *vs* ST5 and RC5 *vs* RT5 included 1445 (63.41%) and 1177 (49.06%) up-regulated genes, 834 (36.59%) and 1222 (50.94%) down-regulated genes, respectively. In SC7 *vs* ST7 and RC7 *vs* RT7, 2358 and 1447 DEGs were identified, respectively.

In the S line, out of 2358 differentially expressed genes (DEGs), 591 (25.06%) showed increased expression while 1767 (74.94%) exhibited decreased expression. The 1447 DEGs in the R line contained 485 up-regulated genes (33.52%) and 962 down-regulated genes (66.48%). In general, with longer times of Al stress, the proportion of up-regulated genes in S and R lines decreased, while the proportion of down-regulated genes increased. Finally, on the 7th day of treatment, the proportion of down-regulated DEGs in the S line surpassed that in the R line, reinforcing that the S line exhibited greater sensitivity to Al stress at the molecular scale.

As illustrated in the **Supplementary Figures S1A, B**, a total of five genes were co-expressed across the six groups. Notably, the gene encoding F-box protein (*BnaC05g35820D*) exhibited up-regulation in all groups. Conversely, genes encoding the peroxidase protein (*BnaC03g33490D*) and lipid transporter (*BnaC07g13240D*) showed consistent down-regulation across all groups. The gene encoding ethylene response factor 104 (*BnaA03g40380D*) demonstrated both up-regulation and down-regulation in both R and S lines. Additionally, a gene encoding pyruvate decarboxylase protein (*BnaAnng13920D*) was observed to be down-regulated on the 3rd, 5th, and 7th days in the R line, and on the 3rd and 5th days in S line, but it was up-regulated on 7th day in S line. These genes were associated with protein degradation, redox reactions, plant hormone signal transduction, and energy metabolism. It is evident that both the S and R lines are capable of activating these associated functional genes in response to Al stress.

### 3.3 Screening for DEGs related to aluminum stress signal transduction

**Figure 3:**
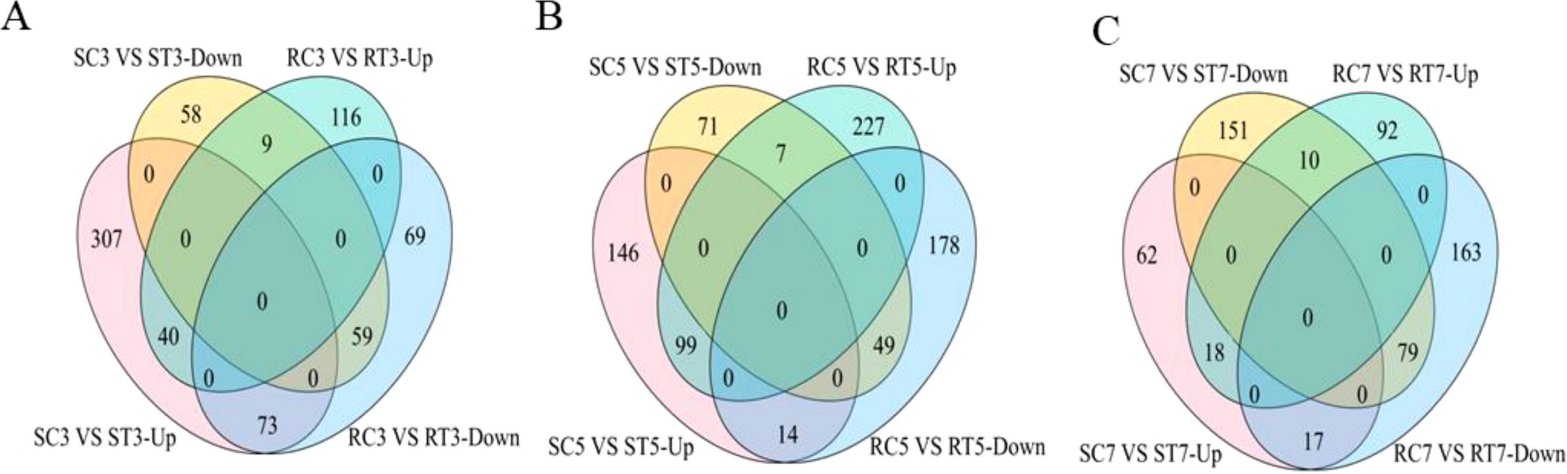
Venn diagrams showing the number of DEGs related to Al stress signal transduction pathways between S and R lines at each stage of Al treatment. **(A–C)** VEEN diagrams related to the number of DEGs on 3rd, 5th and 7th day of aluminum treatment, respectively.

The expression of differential multiples (log2 FC) of DEGs was mapped to the Al stress signal transduction pathway using MapMan software. A total of 1,036 DEGs were screened for adaptation to Al stress (**Supplementary Table S2**). By the third day of Al exposure, the majority of differentially expressed genes (613 out of 820) in the S line were upregulated due to Al stress, whereas in the R line, only about half (234 out of 521) showed increased expression (**Figure 3**; **Supplementary Table S3**). On the fifth day, more than two-thirds of DEGs (457 out of 653) in the S line were upregulated, while in the R line 50% of the genes (424 out of 807) were found to be upregulated. On the seventh day, however, only 25% of the DEGs (170 out of 692) showed increased expression in the S line, while 30% of R line genes were upregulated (**Figure 3**; **Supplementary Table S3**). In summary, the S line exhibited a greater quantity of up-regulated DEGs linked to Al stress signal transduction pathway on the 3rd day compared to the 5th and 7th days. The S line demonstrated greater sensitivity to Al stress than the R line, with stronger response observed on the 3rd day. More genes were up-regulated to respond to damage from Al stress on day 3. Similarly, following filtration through the Al stress signal transduction pathway, two genes (*BnaC03g33490D* and *BnaA03g40380D*) were found to be co-expressed across six groups (**Supplementary Figures S1C, D**). These genes are associated with redox reactions and plant hormone signal transduction, respectively.

### 3.4 Screening of hub genes by WGCNA

**Figure 4:**
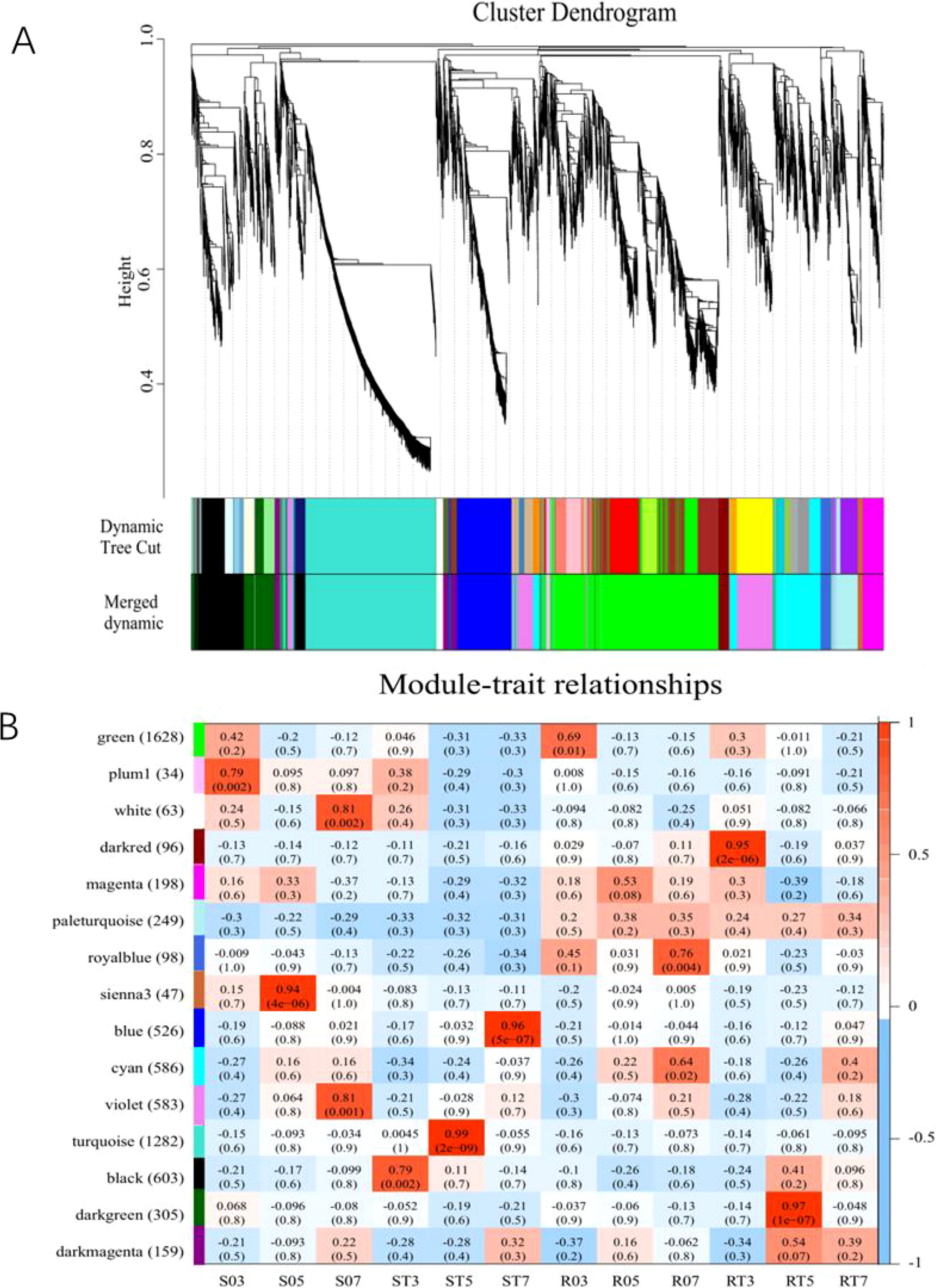
Cluster module tree diagram and module character association diagram. **(A)** Gene cluster number and module cutting of gene co-expression network. Each branch of the gene cluster tree corresponds to a module. **(B)** Heatmap of gene co-expression network module and differential processing. The leftmost color bar indicates different co-expression modules. The numbers in the figure show the correlation between modules and different processes, while the numbers in parentheses indicate the correlation *p* value.

The co-expression network of DEGs between S and R lines was constructed using weighted gene co-expression network analysis (WGCNA), resulting in a co-expression network consisting of 15 distinct modules (**Figure 4A**). The number of genes per module varied from 34 to 1,628. Each module was labeled with different color, and there were 1,708 genes that were not assigned to any modules.

After the co-expression network was utilized to analyze the correlation between different treatments and modules, the correlation heatmap was generated (**Figure 4B**). In this study, five specific modules were identified. Within the S line, the black module containing 603 genes, showed a positive correlation with ST3 (r = 0.79, *p* = 0.002); the turquoise module, which includes 1,282 genes, exhibited a strong positive association with ST5 (r = 0.99, *p* = 2e-09); and the blue module, comprising 526 genes, was positively linked to ST7 (r = 0.96, *p* = 5e-07).

For the R line, the dark red module (96 genes) and the dark green module (305 genes) exhibited significant positive correlations with RT3 (r = 0.95, *p* = 2e-06) and RT5 (r = 0.97, *p* = 1e-07), respectively. By screening the characteristic gene connectivity and gene significance values of the module genes, a total of 321 hub genes were identified in the black module, 983 in the turquoise module, 413 in the blue module, 53 in the dark red module, and 159 in the dark green module (**Supplementary Table S4**).

### 3.5 Screening of key genes

#### 3.5.1 Screening of key genes by combination with WGCNA and QTL

**Figure 5:**
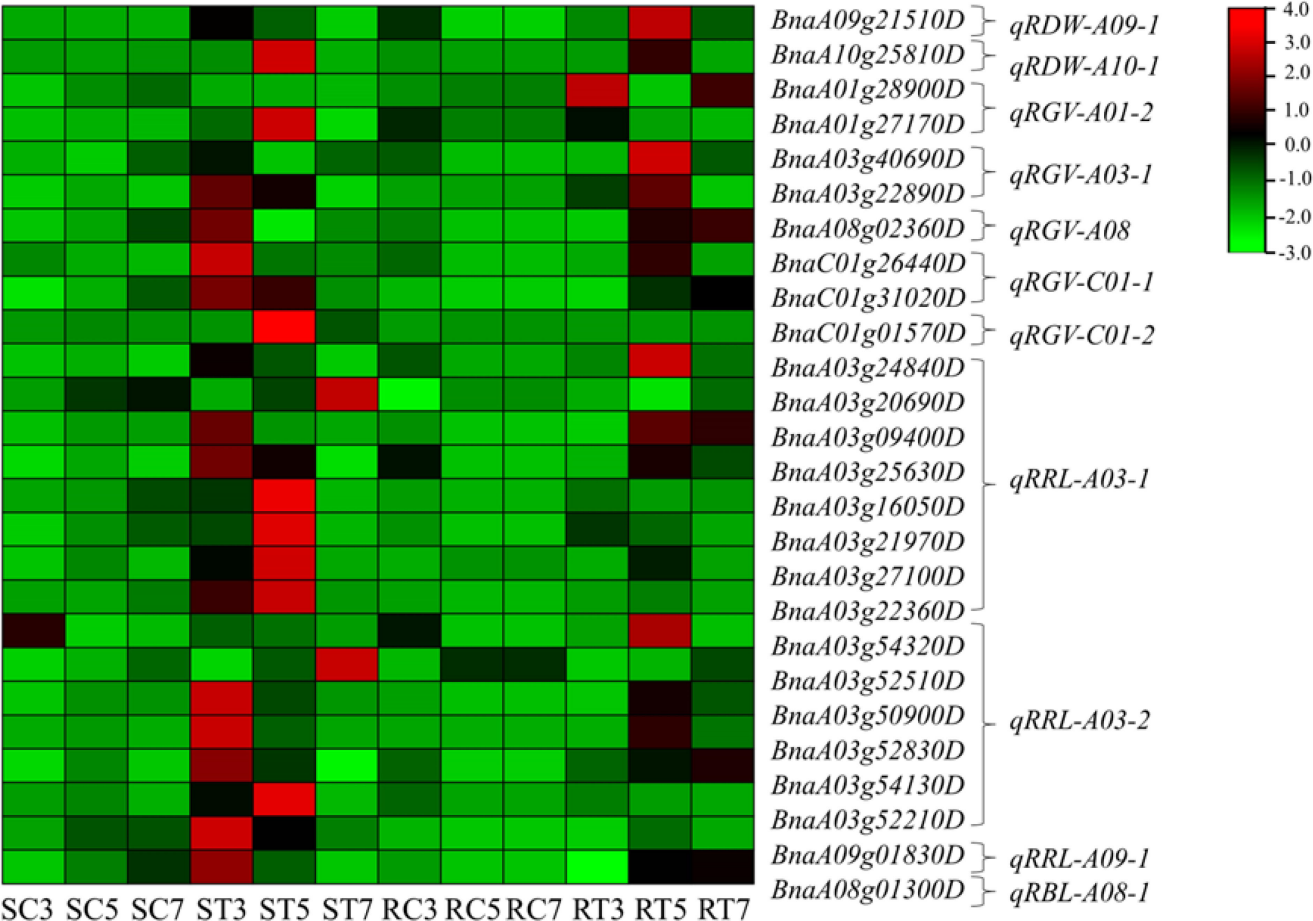
Expression heatmap of candidate key genes. Notes: RDW, relative dry weight; RGV, relative germination vigor; RRL, relative root length; and RBL, relative bud length. qRDW-A09-1 is the first QTL of relative dry weight on the A09 chromosome. In addition, based on the average gene expression level of the same sample, expression levels higher than the average are considered positive value and marked in red; On the contrary, if the expression level is lower than the average, it is negative value and marked in green. The depth of color indicates the degree of difference between gene expression levels and the mean.

By combining DEGs related to Al stress signal transduction pathways and module hub genes, a total of 332 candidate genes were identified (**Supplementary Table S5**). These candidate genes were mapped to QTL intervals of related traits in rapeseed germination under Al stress, resulting in the identification of 26 key genes (**Figure 5**). Among these, eight genes were located at five QTLs: namely *qRGV-A01-2*, *qRGV-A03-1*, *qRGV-A08*, *qRGV-C01-1* and *qRGV-C01-2*, and they were related to RGV. There were 15 genes associated with RRL, located at three QTLs: *qRRL-A03-1*, *qRRL-A03-2* and *qRRL-A09-1*. Two genes related to the RDW were found at two QTLs: *qRDW-A09-1* and q*RDW-A10-1*. The final gene was associated with RBL and was located at *qRBL-A08-1.*

#### 3.5.2 Functional analysis of key genes

The 26 key genes had functions related to plant cell wall modification, hormone signal transduction, stress proteins, redox balance, calcium signal transduction, and transcriptional regulation (**Supplementary Table S6**) and respond to external stimuli through intracellular or intercellular messages. The first type of gene is related to plant hormone signal transduction: *BnaC01g01570D* encodes the BRU6 protein, *BnaA08g01300D* and *BnaA03g52830D* encode ERF8 and ERF043, and *BnaA01g27170D* encodes JAZ3, which are involved in auxin, ethylene, and jasmonic acid signal transduction, respectively. The second type of gene is involved in cell wall modification: *BnaA03g54320D* encodes xylan endoglucosyltransferase/hydrolase 7 (XTH7) and is linked to β-glucanase. The third type of gene is associated with stress proteins: *BnaA03g25630D* and *BnaA08g02360D* are involved in proteolysis and encode F-box and U-box protein, respectively. The fourth type of gene is connected with redox balance and is represented by *BnaA03g52510D*, which encodes peroxidase (POD), and *BnaA03g21970D*, which encodes glutathione transferase 5 (GSTU5). The fifth type of gene is part of the calcium signal transduction system, including the calmodulin family protein encoded by *BnaA03g50900D*. The last type includes transcription factors such as *BnaA03g40690D* and *BnaA03g52830D*, which encode RRTF1; *BnaC01g26440D* and *BnaC01g31020D* which encode MYB77 and MYB15, respectively; *BnaA03g22890D*, *BnaA03g24840D*, and *BnaA09g21510D*, which encode HSFA2 and HSFB2B, both of which are associated with heat-shock proteins.

#### 3.5.3 Expression of key genes in S and R lines

**Figure 6:**
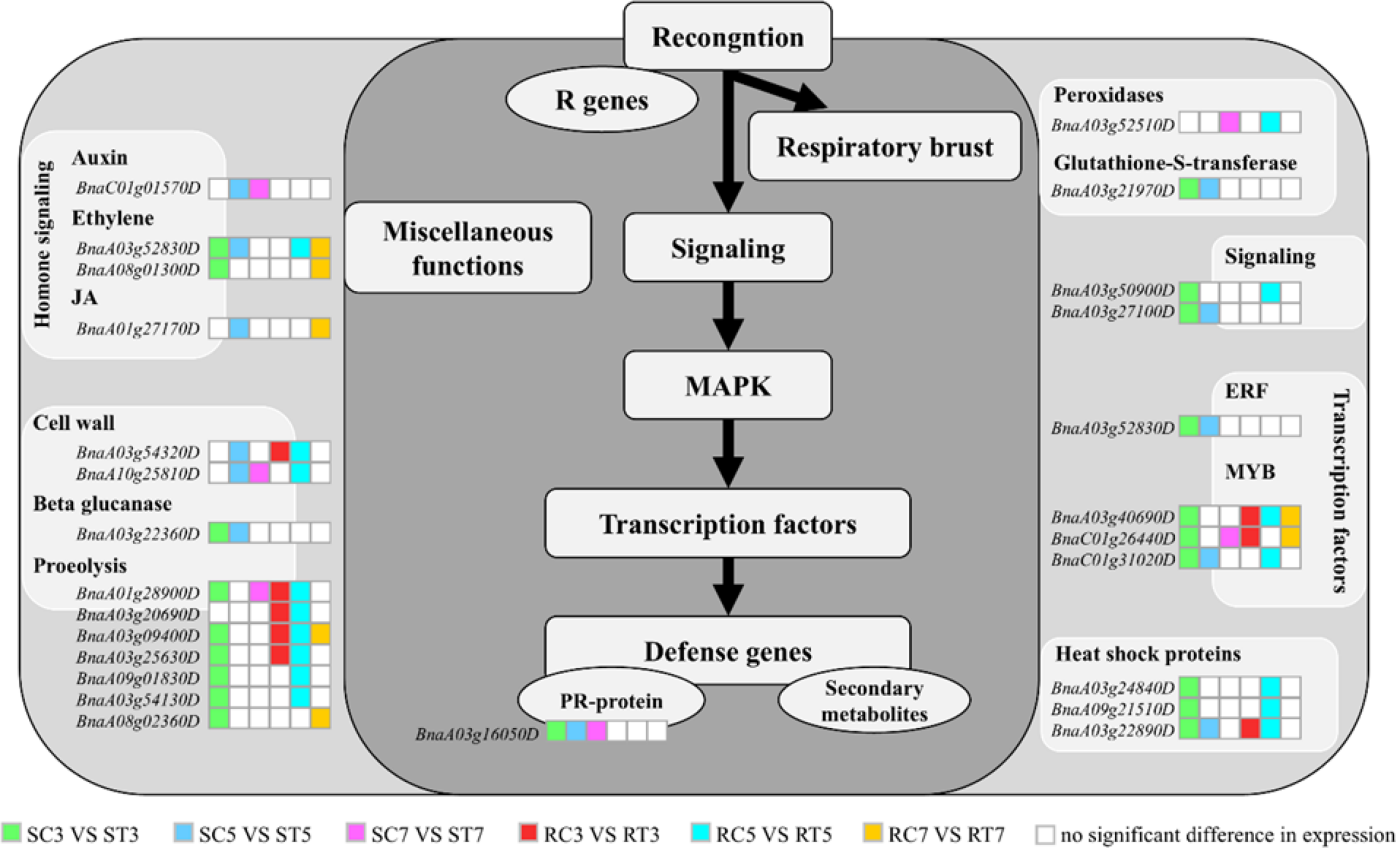
Enrichment analysis of differentially expressed genes in S and R signal transduction pathways under aluminum stress. Different colored blocks represent different groups, while white blocks indicate non-significant differences in gene expression between groups.

Following the enrichment analysis of crucial genes in the Al stress signal transduction pathway (**Figure 6**), the S line exhibited a greater number of essential genes related to ethylene signaling, proteolysis, calcium signaling, transcription factor regulation, and heat-shock protein production compared to the R line, particularly on days 3 and 5. The gene upregulation in the R line mainly occurred on the 5th day after Al treatment, indicating a delayed response to Al stress. It can be inferred that the S line responds to stress earlier than the R line by upregulating genes related to calcium, transcription factors, oxidoreductases, cell walls integrity, and plant hormones to alleviate Al stress.

**Figure 7:**
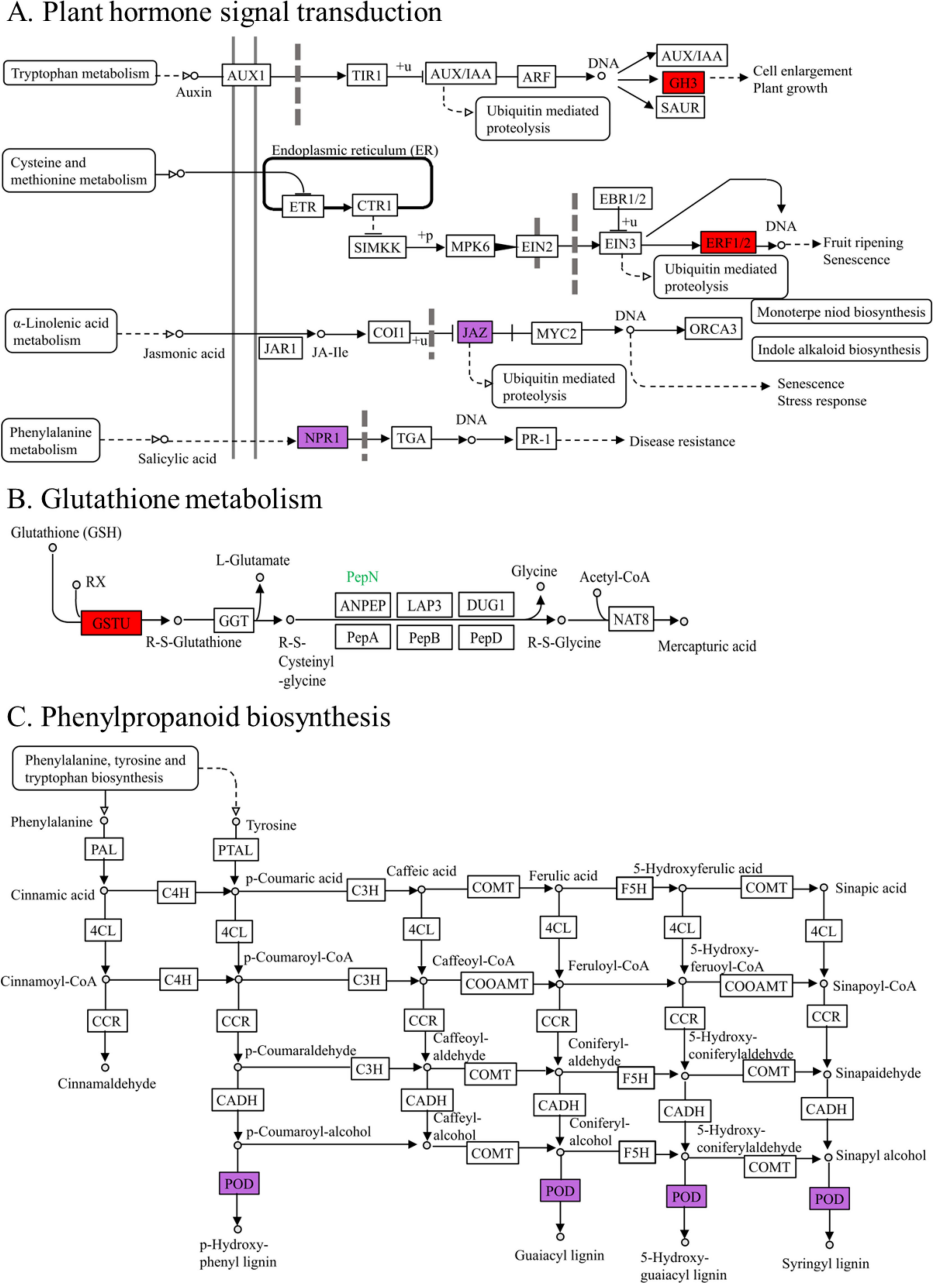
Metabolic pathways in which key genes related to aluminum toxicity stress are involved. The red box represents up-regulated genes in the S line or in the R line. The purple boxes show up-regulated and down-regulated genes in the S and R lines. **(A)** Plant hormone signal transduction. **(B)** Glutathione metabolism. **(C)** Phenylpropanoid biosynthesis.

By examining the metabolic pathways of crucial genes, it was found that six genes were concentrated in the KEGG metabolic pathway, associated with plant hormone signal transduction, phenylpropanoid biosynthesis, and glutathione metabolism (**Figure 7**). Within the signal transduction pathways of plant hormones (**Figure 7A**), the primary gene associated with auxin signaling (*BnaC01g01570D*) was exclusively upregulated in the S line. ERF8 (*BnaA08g01300D*), a key transcription factor related to ethylene signal transduction, was upregulated in both S and R lines. The principal gene associated with jasmonic acid (*BnaA01g27170D*) showed increased expression in the S line and downregulated in the R line. Within the glutathione metabolism pathway (**Figure 7B**), an important gene associated with glutathione S-transferase (GST) (*BnaA03g21970D*) showed increased expression in the S line. In the phenylpropanoid biosynthesis pathway (**Figure 7C**), the crucial gene associated with peroxidase (POD) (*BnaA03g52510D*) exhibited increased expression in the S line but decreased expression in the R line. SAM-MTase showed significant similarity to caffeic acid 3-O-methyltransferase (COMT), and the gene *BnaA10g25810D*, which encodes SAM-MTase, exhibited both increased and decreased expression in the S line, while it was solely up-regulated in the R line. Therefore, the S line can withstand Al stress by increasing the expression of crucial genes associated with redox equilibrium and hormone signaling in plants.

### 3.6 qRT-PCR verification

**Figure 8:**
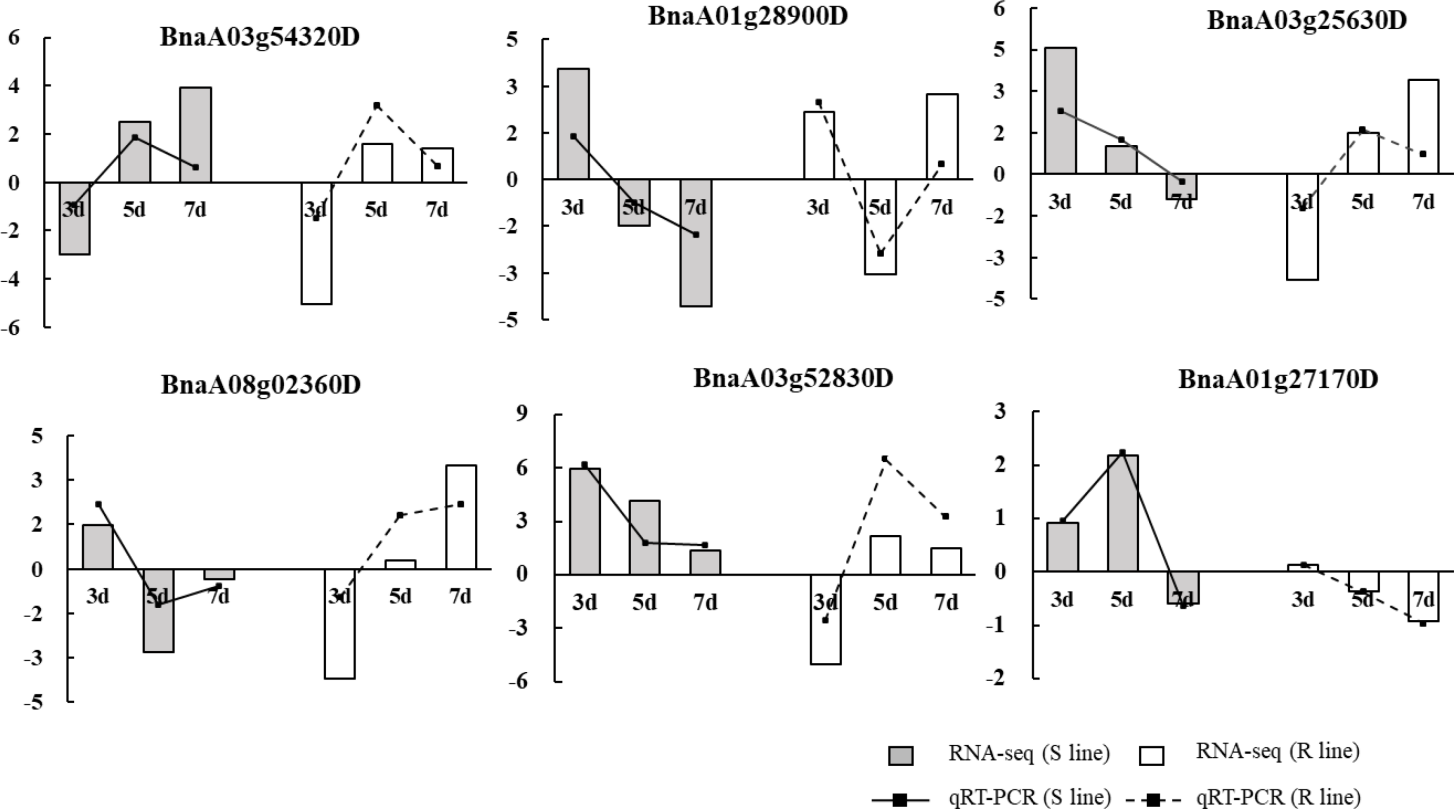
Validation of RNA-seq data by qRT-PCR. Six key genes were selected for validation, and they showed a similar tendency to the RNA-seq results. The y-axis showed the fold-changes of three treatment stages compared to the beginning point, with positive values indicating up-regulation and negative values indicating down-regulation.

In order to test the RNA-seq data, six key genes were selected for qRT-PCR verification based on the expression patterns of observed at three time points of the S and R lines. The results indicated that the expression patterns from qRT-PCR and RNA-seq showed similar up- and down-regulation, suggesting that the RNA-seq data obtained in this research have high reliability (**Figure 8**).

## 4 Discussion

The transcriptome data obtained through high-throughput sequencing can provide valuable information for investigating gene expression and functional genomics of rapeseed under Al stress. MapMan software was used to screen the DEGs involved in Al stress signal transduction, significantly narrowing down the range of candidate genes for further analysis. WGCNA provided a time series analysis of the gene expression response in rapeseed under Al stress, which was essential for determining the functions of genes related to resistance to Al stress. The co-expressed gene modules associated with these phenotypes were identified using eigengene network methods, and the putative major functional genes for Al tolerance were predicted. QTL analysis is an effective method for pinpointing genes linked to complex traits. To identify the genes responsible for the impact of Al stress on rapeseed germination, differentially expressed genes (DEGs) associated with Al stress signaling and central genes in WGCNA modules were cross-referenced with candidate genes within the QTL confidence interval, resulting in the identification of 26 crucial genes. These findings are significant for understanding the molecular basis of Al tolerance in rapeseed and for the effective utilization of germplasm resources. Through comprehensive screening of key genes, the potential loss of candidate genes was avoided, leading to the acquisition of meaningful information.

### 4.1 The aluminum tolerance of rapeseed can be regulated by the expression of transcription factors

The regulation of plant adaptation to Al stress involves numerous transcription factors, such as AP2/ERF, MYB, bHLH, as well as the zinc finger and WRKY families (Huang et al., 2021). The key transcription factors screened in this study included ERF, RRTF, MYB, and HSF (**Supplementary Table S6**). The AP2/ERF transcription factor, characterized by its AP2 domain, is crucial for plant responses to various environmental stresses, including cold, drought, extreme heat, high salinity, and low oxygen levels (Ma et al., 2024). This research identified RRTF1 (*BnaA03g52830D*), ERF8 (*BnaA08g01300D*), and ERF043 (*BnaA03g52210D*), all of which are members of the AP2/ERF transcription factor family. MYB primarily acts as a transcriptional activator in response to both biotic and abiotic stresses (Chen et al., 2018). Additionally, MYB77, encoded by *BnaA03g40690D* and *BnaC01g26440D*, and MYB15, encoded by *BnaC01g31020D*, were also screened. Studies have shown that MYB77 is mainly involved in auxin responses and reactive oxygen species (ROS) clearance (Sng et al., 2019), while MYB15 participates in lignin synthesis (Kim et al., 2020). Both ROS removal and lignin synthesis have been shown to be part of the response to Al stress (Sun et al., 2020).

The increase in MYB expression triggered by aluminum could enhance rapeseed’s resistance to Al stress by activating genes that confer Al tolerance. In addition, two heat-shock transcription factors, AT-HSFB2B and HSFA2, were screened in this study (**Supplementary Table S6**). Research has indicated that various HSFs can work together to control abiotic stress in plants (Ikeda et al., 2011; Nishizawa-Yokoi et al., 2011), suggesting that HSFs might improve rapeseed’s response to Al stress. However, the regulatory mechanism of HSFs under Al stress has not yet been studied.

### 4.2 Aluminum stress can affect root length in rapeseed through plant hormones

Exposure to stress causes plants to alter their hormone levels, triggering a cascade of physiological and biochemical responses to cope with the stress (Waadt et al., 2022). Aluminum can alter the levels and placement of auxin within plants, leading to its movement from the remote transition area of the roots to the elongation region, thereby decreasing root growth (Wang et al., 2023). The BRU6 protein encoded by *BnaC01g01570D*, a key gene screened in this study, is part of the auxin signal transduction pathway. It was up-regulated on the 5th and 7th days in the S line under Al treatment but did not show differential expression in the R line. Therefore, it can be inferred that the synthesis of auxin may inhibit root growth and shorten root length in the S line under Al stress. The R line was less affected by auxin and showed little change in root length. This is also evident in the variations in root length between the S and R lines (**Figure 1**). Sun et al. (2010) found that *Arabidopsis* rapidly released ethylene under Al stress, resulting in the redistribution of auxin to the roots, thus inhibiting their growth. In this study, the gene *BnaA08g01300D*, which encodes ERF8—a transcription factor associated with ethylene signal transduction—was up-regulated on day 3 in the S line under Al treatment, and on day 7 in the R line. Thus, it is plausible to infer that Al stress hindered the root development of the S line during the initial phase of germination, whereas the R line experienced root growth inhibition during the middle and later phases. Similar characteristics can be observed in **Figure 1**. The findings revealed that the suppression of root growth was governed by COI1-dependent jasmonic acid pathways, influenced by ethylene and not reliant on auxin signaling (Yang et al., 2017). The crucial gene *BnaA01g27170D* encodes JAZ3, which plays a role in the jasmonic acid signaling pathway. This gene was up-regulated on the 5th day in the S line under Al treatment and down-regulated on the 7th day in the R line. This expression pattern is similar to that of the auxin signal transduction gene, *BnaC01g01570D*. The findings suggest that, due to plant hormones, Al stress more severely restricts root length in the S line compared to the R line.

### 4.3 Stress protein functions for resisting aluminum stress in rapeseed during germination

Aluminum toxicity can induce the production of endogenous hormones (auxin, ethylene, jasmonic acid) in plants, thereby causing protein ubiquitination (**Figure 7**). Protein ubiquitination is a vital pathway mediating plant protein degradation and contributing to seed dormancy, germination, and the abiotic stress response (Xu and Xue, 2019). The U-box protein is an important component in the process of protein ubiquitination. In this study, the expression of *BnaA08g02360D*, which encodes the RING/U-box protein, was upregulated on day 3 in the S line with aluminum (Al) treatment, and on day 7 in the R line. The results showed that the S line responded to Al stress earlier than the R line. In *Arabidopsis thaliana*, the RING type E3 ligase coding gene, *At2g34000*, was up-regulated at 6 and 48 h after Al treatment (Kumari et al., 2008). An important gene, *BnaA03g25630D*, codes for the F-box protein, which is involved in regulating the ubiquitin-proteasome system within the SCF complex (Jia et al., 2017). Zhang et al. (2019) isolated and identified an F-box protein, RAE1, in *Arabidopsis thaliana*. STOP1, a transcription factor associated with the malic acid transporter, can bind to the promoter of the RAE1 gene and increase its activity, thereby facilitating the ubiquitination and breakdown of STOP1 via the ubiquitin-proteasome system, boosting resistance to Al toxicity. It may be that rapeseed can respond to Al stress by up-regulating genes involved in protein ubiquitination and degradation, as was also found in *Liriodendron* (Wang et al., 2021c).

The mechanism of plant aluminum (Al) exclusion primarily involves the excretion of organic acid (OA) anions (such as malate, citrate, and oxalate) by roots, thereby preventing Al^3+^ from binding to root cells. These OAs are secreted via root transporter proteins, specifically ALMT (aluminum-activated malate transporter) and MATE (multidrug and toxic compound extrusion) (Bian et al., 2013). In *Arabidopsis thaliana*, the C2H2 transcription factor STOP1 induces the expression of a series of genes, including AtALMT1, promoting malic acid secretion to chelate Al^3+^ and mitigate Al toxicity (Kobayashi et al., 2013). Ligaba et al. (2006) identified *BnALMT1* and *BnALMT2* in rapeseed, which encode aluminum-induced malic acid transporters that facilitate the expulsion of Al^3+^, thus reducing Al toxicity. The MATE family extrudes Al^3+^ by forming aluminum-citrate complexes, thereby alleviating Al stress (Bian et al., 2013). However, this study did not identify any genes related to malic acid or citric acid transport, indicating certain limitations.

### 4.4 Rapeseed alleviates aluminum stress through cell wall modification

Plants can reduce the content of Al in cells by inhibiting the binding of Al^3+^ to the cell wall, which is thought to be a key mechanism of Al tolerance. Changes in cell wall structure mediated by cell wall-modifying enzymes, such as xyloglucan endotransglucosylase/hydrolase (XTH) and cellulase (Sun et al., 2020), can reduce the binding of Al^3+^. XTH is mainly responsible for cutting and reconnecting the xyloglucan chain between microfibrils. The reduction of xyloglucan content leads to plant cell expansion and cell wall relaxation, thus affecting the absorption of Al^3+^ and increasing Al resistance (Bian et al., 2013).

This research identified the primary gene XTH7 (*BnaA03g54320D*), and its expression showed no significant change on the 3rd day of S line Al exposure, but it was up-regulated by the 5th day. In the R line, however, the expression was down-regulated on the 3rd day and up-regulated on the 5th day. The findings indicated that the R line exhibited a delayed response to Al stress and increased expression of the XTH7 gene during the intermediate phases of germination. The glycosylhydrolase (*BnaA03g22360D*) screened in this study is a type of cellulase, and its expression may also influence germination. Overall, these findings support the theory that gene expression controlling cell wall composition is crucial for safeguarding plant cells against stress (Cronmiller et al., 2019).

### 4.5 The up-regulation of antioxidant genes can improve the resistance to aluminum stress in rapeseed

Aluminum can cause plant roots to generate excessive reactive oxygen species, leading to lipid peroxidation and harm to root cell membranes. Plants possess antioxidant enzymes like peroxidase (POD) and non-enzymatic systems such as glutathione S-transferase (GST), which help eliminate oxygen free radicals and mitigate membrane peroxidation damage (Kumari et al., 2018). GST binds toxic substances to glutathione and forms glutathione conjugates, which are transported to vacuoles via ABC transporters (Vaish et al., 2020). POD can remove excess H_2_O_2_ and peroxides from the cells (Sun et al., 2020), thus protecting them from oxidative damage. Under Al stress, the POD gene in rapeseed can be upregulated to reduce ROS levels and alleviate stress damage. In this study, *BnaA03g21970D*, a key gene encoding GSTU5, was upregulated on the 3rd and 5th day after S line Al treatment, while the differential expression was not significant in the R line. The key gene encoding POD, *BnaA03g52510D*, was up-regulated on the seventh day of S line Al treatment but down-regulated on the fifth day in the R line. Therefore, we conclude that the expression of antioxidant genes contributes to the effective defense against Al toxicity, which has also been reported in *A. thaliana* (Kumari et al., 2008), maize (Mattiello et al., 2010), and rice (Awasthi et al., 2019).

### 4.6 Calcium ion signaling plays a role in how rapeseed reacts to aluminum stress

Ca^2+^ (calcium ions) plays a crucial role as a secondary messenger in cellular processes, particularly in how plants respond to Al stress (Chen et al., 2024). The rice genotype with increased tolerance improved its resistance to Al toxicity by up-regulating DEGs related to calcineurin binding protein (Tyagi et al., 2020). In a transcriptome analysis of *Stylosanthes* roots, Al^3+^ interfered with the expression of 21 Ca^2+^ signaling factors, among which 3 differentially expressed genes (DEGs) were up-regulated (Jiang et al., 2018). Aluminum stress triggers the CML protein in the root tip transition zone, leading to inhibited root growth (Zhu et al., 2022). In this study, BnaA03g50900D, which encodes the CaLB protein, and BnaA03g27100D, which encodes α/β hydrolase superfamily proteins, were also screened (Suplatov et al., 2012); both were related to calcium signaling pathways and up-regulated in both S and R lines. Therefore, various Ca^2+^ signaling mechanisms collectively enhance rapeseed’s resistance to Al stress.

## 5 Conclusions

In this study, 26 key genes were identified through the combination of RNA-seq, WGCNA, and QTL analysis. Their roles primarily encompassed transcriptional regulation, synthesis of stress proteins, redox homeostasis, plant hormone signaling, cell wall alteration, and calcium signaling. The analysis of the metabolic pathways of these essential genes revealed that they were predominantly involved in plant hormone signaling, glutathione metabolism, and phenylpropanoid synthesis. Overall, the S line exhibits greater sensitivity to Al stress and responds earlier to this stress compared to the R line. These findings provide a crucial empirical foundation for advancing research on Al resistance mechanisms and developing aluminum-tolerant varieties.

## Data Availability

The original contributions presented in the study are publicly available. This data can be found at the National Center for Biotechnology Information (NCBI) using accession number PRJNA1134627; further inquiries can be directed to the corresponding author.

## References

[B1] Awasthi J. P. Saha B. Panigrahi J. Yanase E. Koyama H. Panda S. K. (2019). Redox balance, metabolic fingerprint and physiological characterization in contrasting North East Indian rice for Aluminum stress tolerance. Sci. Rep-Uk 9, 8681. doi: 10.1038/s41598-019-45158-3 PMC6581886 31213660

[B2] Bian M. Zhou M. Sun D. Li C. (2013). Molecular approaches unravel the mechanism of acid soil tolerance in plants. Crop J. 1, 91–104. doi: 10.1016/j.cj.2013.08.002

[B3] Chen C. Chen H. Zhang Y. Thomas H. R. Frank M. H. He Y.. (2020). TBtools: an integrative toolkit developed for interactive analyses of big biological data. Mol. Plant 13, 1194–1202. doi: 10.1016/j.molp.2020.06.009 32585190

[B4] Chen J. S. Wang S. T. Mei Q. Sun T. Hu J. T. Xiao G. S.. (2024). The role of CBL–CIPK signaling in plant responses to biotic and abiotic stresses. Plant Mol. Biol. 114, 53. doi: 10.1007/s11103-024-01417-0 38714550

[B5] Chen Y. H. Cao Y. Y. Wang L. J. Li L. M. Yang J. Zou M. X. (2018). Identification of *MYB* transcription factor genes and their expression during abiotic stresses in maize. Biol. Plantarum 62, 222–230. doi: 10.1007/s10535-017-0756-1

[B6] Cronmiller E. Toor D. Shao N. C. Kariyawasam T. Wang M. H. Lee J. (2019). Cell wall integrity signaling regulates cell wall-related gene expression in *Chlamydomonas reinhardtii* . Sci. Rep-Uk 9, 12204. doi: 10.1038/s41598-019-48523-4 PMC6704257 31434930

[B7] Gao H. Ye S. Wu J. Wang L. Wang R. Lei W.. (2021). Genome-wide association analysis of aluminum tolerance related traits in rapeseed (*Brassica napus* L.) during germination. Genet. Resour Crop Evol. 68, 335–357. doi: 10.1007/s10722-020-00989-2

[B8] Geng L. Zhang W. Zou T. Du Q. Ma X. Cui D.. (2023). Integrating linkage mapping and comparative transcriptome analysis for discovering candidate genes associated with salt tolerance in rice. Front. Plant Sci. 14. doi: 10.3389/fpls.2023.1065334 PMC9904508 36760644

[B9] Huang D. Gong Z. Chen X. Wang H. Tan R. Mao Y. (2021). Transcriptomic responses to aluminum stress in tea plant leaves. Sci. Rep. 11, 5800. doi: 10.1038/s41598-021-85393-1 33707704 PMC7952733

[B10] Huang G. Ma S. Bai L. Zhang L. Ma H. Jia P.. (2012). Signal transduction during cold, salt, and drought stresses in plants. Mol. Biol. Rep. 39, 969–987. doi: 10.1007/s11033-011-0823-1 21573796

[B11] Ikeda M. Mitsuda N. Ohme-Takagi M. (2011). *Arabidopsis* HsfB1 and HsfB2b act as repressors of the expression of heat-inducible Hsfs but positively regulate the acquired thermotolerance. Plant Physiol. 157, 1243–1254. doi: 10.1104/pp.111.179036 21908690 PMC3252156

[B12] Jia Q. Xiao Z. X. Wong F. L. Sun S. Liang K. J. Lam H. M. (2017). Genome-wide analyses of the soybean F-box gene family in response to salt stress. Int. J. Mol. Sci. 18, 818. doi: 10.3390/ijms18040818 28417911 PMC5412402

[B13] Jiang C. Liu L. Li X. Han R. Wei Y. Yu Y. (2018). Insights into aluminum-tolerance pathways in *Stylosanthes* as revealed by RNA-Seq analysis. Sci. Rep-Uk 8, 6072. doi: 10.1038/s41598-018-24536-3 PMC5904178 29666506

[B14] Kalve S. House M. A. Tar’An B. (2023). Freezing stress response of wild and cultivated chickpeas. Front. Plant Sci. 14. doi: 10.3389/fpls.2023.1310459 PMC10876003 38375446

[B15] Kim S. H. Lam P. Y. Lee M. H. Jeon H. S. Tobimatsu Y. Park O. K. (2020). The *arabidopsis* R2R3 MYB transcription factor MYB15 is a key regulator of lignin biosynthesis in effector-triggered immunity. Front. Plant Sci. 11. doi: 10.3389/fpls.2020.583153 PMC7527528 33042196

[B16] Kobayashi Y. Kobayashi Y. Sugimoto M. Lakshmanan V. Iuchi S. Kobayashi M.. (2013). Characterization of the complex regulation of *AtALMT1* expression in response to phytohormones and other inducers. Plant Physiol. 162, 732–740. doi: 10.1104/pp.113.218065 23624855 PMC3668066

[B17] Kochian L. V. Hoekenga O. A. Pineros M. A. (2004). How do crop plants tolerate acid soils? Mechanisms of aluminum tolerance and phosphorous efficiency. Annu. Rev. Plant Biol. 55, 459–493. doi: 10.1146/annurev.arplant.55.031903.141655 15377228

[B18] Kumari M. Taylor G. J. Deyholos M. K. (2008). Transcriptomic responses to aluminum stress in roots of *Arabidopsis thaliana* . Mol. Genet. Genomics 279, 339–357. doi: 10.1007/s00438-007-0316-z 18270741

[B19] Kumari N. Yadav M. Sharma V. (2018). Differential response of *Brassica juncea* cultivars to Al; consequences for chlorophyll a fluorescence, antioxidants and *psb A* gene. J. Plant Interact. 13, 496–505. doi: 10.1080/17429145.2018.1526980

[B20] Langfelder P. Horvath S. (2008). WGCNA: an R package for weighted correlation network analysis. BMC Bioinform. 9, 559. doi: 10.1186/1471-2105-9-559 PMC2631488 19114008

[B21] Li Y. Ye H. Song L. Vuong T. D. Song Q. Zhao L.. (2021). Identification and characterization of novel QTL conferring internal detoxification of aluminium in soybean. J. Exp. Bot. 72, 4993–5009. doi: 10.1093/jxb/erab168 33893801

[B22] Ligaba A. Katsuhara M. Ryan P. R. Shibasaka M. Matsumoto H. (2006). The BnALMT1 and BnALMT2 genes from rape encode aluminum-activated malate transporters that enhance the aluminum resistance of plant cells. Plant Physiol. 142, 1294–1303. doi: 10.1104/pp.106.085233 17028155 PMC1630743

[B23] Liu H. Zhu R. Shu K. Lv W. Wang S. Wang C. (2022). Aluminum stress signaling, response, and adaptive mechanisms in plants. Plant Signal Behav. 17, 2057060. doi: 10.1080/15592324.2022.2057060 35467484 PMC9045826

[B24] Ma L. An R. Jiang L. Zhang C. Li Z. Zou C. (2022). Effects of *ZmHIPP* on lead tolerance in maize seedlings: Novel ideas for soil bioremediation. J. Hazard Mater 430, 128457. doi: 10.1016/j.jhazmat.2022.128457 35180524

[B25] Ma J. F. Furukawa J. (2003). Recent progress in the research of external Al detoxification in higher plants: a minireview. J. Inorg Biochem. 97, 46–51. doi: 10.1016/S0162-0134(03)00245-9 14507459

[B26] Ma Z. Hu L. Jiang W. (2024). Understanding AP2/ERF transcription factor responses and tolerance to various abiotic stresses in plants: a comprehensive review. Int. J. Mol. Sci. 25, 893. doi: 10.3390/ijms25020893 38255967 PMC10815832

[B27] Mattiello L. Kirst M. Da Silva F. R. Jorge R. A. Menossi M. (2010). Transcriptional profile of maize roots under acid soil growth. BMC Plant Biol. 10, 196. doi: 10.1186/1471-2229-10-196 20828383 PMC2956545

[B28] Nishizawa-Yokoi A. Nosaka R. Hayashi H. Tainaka H. Maruta T. Tamoi M.. (2011). HsfA1d and HsfA1e involved in the transcriptional regulation of HsfA2 function as key regulators for the Hsf signaling network in response to environmental stress. Plant Cell Physiol. 52, 933–945. doi: 10.1093/pcp/pcr045 21471117

[B29] Panda S. K. Baluska F. Matsumoto H. (2009). Aluminum stress signaling in plants. Plant Signal Behav. 4, 592–597. doi: 10.4161/psb.4.7.8903 19820334 PMC2710549

[B30] Pinto V. B. Vidigal P. M. P. Dal-Bianco M. Almeida-Silva F. Venancio T. M. Viana J. M.S. (2023). Transcriptome-based strategies for identifying aluminum tolerance genes in popcorn (*Zea mays* L. var. *everta*). Sci. Rep-Uk 13, 19400. doi: 10.1038/s41598-023-46810-9 PMC10632369 37938583

[B31] Sami A. Shah F. A. Abdullah M. Zhou X. Yan Y. Zhu Z.. (2020). Melatonin mitigates cadmium and aluminium toxicity through modulation of antioxidant potential in *Brassica napus* L. Plant Biol. (Stuttg) 22, 679–690. doi: 10.1111/plb.13093 32003103

[B32] Sara F. Barat A. F. Nafiseh M. N. Sirous T. Abbas M. Bahram H. (2020). Mapping QTLs associated with grain yield and yield-related traits under aluminum stress in bread wheat. Crop Pasture Sci. 71, 429–444. doi: 10.1071/CP19511

[B33] Smith B. E. Kemmer S. Decker S. Lu C. (2024). Quantitative trait locus (QTL) mapping and transcriptome profiling identify QTLs and candidate genes associated with heat stress response during reproductive development in *Camelina sativa* . Food Energy Secur 13, e531. doi: 10.1002/fes3.531

[B34] Sng N. J. Kolaczkowski B. Ferl R. J. Paul A. (2019). A member of the CONSTANS-Like protein family is a putative regulator of reactive oxygen species homeostasis and spaceflight physiological adaptation. Aob Plants 11, y75. doi: 10.1093/aobpla/ply075 PMC6348315 30705745

[B35] Sun C. Lv T. Huang L. Liu X. Jin C. Lin X. (2020). Melatonin ameliorates aluminum toxicity through enhancing aluminum exclusion and reestablishing redox homeostasis in roots of wheat. J. Pineal Res. 68, e12642. doi: 10.1111/jpi.12642 32092171

[B36] Sun P. Tian Q. Y. Chen J. Zhang W. H. (2010). Aluminium-induced inhibition of root elongation in *Arabidopsis* is mediated by ethylene and auxin. J. Exp. Bot. 61, 347–356. doi: 10.1093/jxb/erp306 19858117 PMC2803203

[B37] Suplatov D. A. Besenmatter W. Svedas V. K. Svendsen A. (2012). Bioinformatic analysis of α/β-hydrolase fold enzymes reveals subfamily-specific positions responsible for discrimination of amidase and lipase activities. Protein Eng. Des. Sel 25, 689–697. doi: 10.1093/protein/gzs068 23043134

[B38] Thimm O. Bläsing O. Gibon Y. Nagel A. Meyer S. Krüger P.. (2004). MAPMAN: a user-driven tool to display genomics data sets onto diagrams of metabolic pathways and other biological processes. Plant J. 37, 914–939. doi: 10.1111/j.1365-313x.2004.02016.x 14996223

[B39] Tyagi W. Yumnam J. S. Sen D. Rai M. (2020). Root transcriptome reveals efficient cell signaling and energy conservation key to aluminum toxicity tolerance in acidic soil adapted rice genotype. Sci. Rep-Uk 10, 4580. doi: 10.1038/s41598-020-61305-7 PMC7067865 32165659

[B40] Vaish S. Gupta D. Mehrotra R. Mehrotra S. Basantani M. K. (2020). Glutathione S-transferase: a versatile protein family. 3 Biotech. 10, 321. doi: 10.1007/s13205-020-02312-3 PMC7320970 32656054

[B41] Waadt R. Seller C. A. Hsu P. Takahashi Y. Munemasa S. Schroeder J. I. (2022). Plant hormone regulation of abiotic stress responses. Nat. Rev. Mol. Cell Bio 23, 680–694. doi: 10.1038/s41580-022-00479-6 35513717 PMC9592120

[B42] Wang P. Dong Y. Zhu L. Hao Z. Hu L. Hu X.. (2021c). The role of γ-aminobutyric acid in aluminum stress tolerance in a woody plant, *Liriodendron chinense × tulipifera* . Hortic. Res-England 8, 80. doi: 10.1038/s41438-021-00517-y PMC8012378 33790239

[B43] Wang L. Feng Z. Wang X. Wang X. Zhang X. (2010). DEGseq: an R package for identifying differentially expressed genes from RNA-seq data. Bioinformatics 26, 136–138. doi: 10.1093/bioinformatics/btp612 19855105

[B44] Wang T. Hu Y. Hu C. Tan J. Xu H. Li P.. (2022). Transcriptome analysis of response to aluminum stress in *Pinus massoniana* . Forests 13, 837. doi: 10.3390/f13060837

[B45] Wang P. Wan N. Horst W. J. Yang Z. (2023). From stress to responses: aluminium-induced signalling in the root apex. J. Exp. Bot. 74, 1358–1371. doi: 10.1093/jxb/erac516 36609593

[B46] Wang L. Wang R. Lei W. Wu J. Li C. Shi H. (2021b). Transcriptome analysis reveals gene responses to herbicide, tribenuron methyl, in *Brassica napus* L. during seed germination. BMC Genomics 22, 299. doi: 10.1186/s12864-021-07614-1 33892633 PMC8067372

[B47] Wang R. Wang L. Lei W. Wu J. Shi H. Li C.. (2021a). Screening candidate genes related to aluminum toxicity stress at germination stage via RNA-seq and QTL mapping in *Brassica napus* L. Acta Agron. Sin. 47, 2407–2422. doi: 10.3724/SP.J.1006.2021.04231

[B48] Wang R. Wang L. Ye S. Gao H. Lei W. Wu J.. (2020). QTL mapping of seed germination related traits in *Brassica napus* under aluminum stress. Acta Agron. Sin. 46, 832–843. doi: 10.3724/SP.J.1006.2020.94154

[B49] Xie C. Mao X. Huang J. Ding Y. Wu J. Dong S.. (2011). KOBAS 2.0: a web server for annotation and identification of enriched pathways and diseases. Nucleic Acids Res. 39, W316–W322. doi: 10.1093/nar/gkr483 21715386 PMC3125809

[B50] Xu F. Xue H. (2019). The ubiquitin-proteasome system in plant responses to environments. Plant Cell Environ. 42, 2931–2944. doi: 10.1111/pce.13633 31364170

[B51] Yan L. Riaz M. Wu X. Wang Y. Du C. Jiang C. (2018). Interaction of boron and aluminum on the physiological characteristics of rape (*Brassica napus* L.) seedlings. Acta Physiol. Plant 40, 33. doi: 10.1007/s11738-018-2614-y

[B52] Yang Z. B. He C. Ma Y. Herde M. Ding Z. (2017). Jasmonic Acid enhances Al-induced root growth inhibition. Plant Physiol. 173, 1420–1433. doi: 10.1104/pp.16.01756 27932419 PMC5291015

[B53] Yin M. Zheng Z. Zhang Y. Wang S. Zuo L. Lei Y.. (2024). Identification of key genes and pathways for anaerobic germination tolerance in rice using weighted gene co-expression network analysis (WGCNA) in association with quantitative trait locus (QTL) mapping. Rice 17, 37. doi: 10.1186/s12284-024-00714-y 38819744 PMC11143092

[B54] Zhang Y. Zhang J. Guo J. Zhou F. Singh S. Xu X.. (2019). F-box protein RAE1 regulates the stability of the aluminum-resistance transcription factor STOP1 in *Arabidopsis* . Proc. Natl. Acad. Sci. 116, 319–327. doi: 10.1073/pnas.1814426116 30559192 PMC6320511

[B55] Zhao H. Basu U. Kebede B. Qu C. Li J. Rahman H.. (2019). Fine mapping of the major QTL for seed coat color in *Brassica rapa* var. Yellow Sarson by use of NIL populations and transcriptome sequencing for identification of the candidate genes. PLoS One 14, e209982. doi: 10.1371/journal.pone.0209982 PMC6361427 30716096

[B56] Zhao W. Li J. Deng K. Shi R. Jiang J.. (2020). Effects of crop straw biochars on aluminum species in soil solution as related with the growth and yield of canola (*Brassica napus* L.) in an acidic Ultisol under field condition. Environ. Sci. pollut. R 27, 30178–30189. doi: 10.1007/s11356-020-09330-x 32451890

[B57] Zhu J. (2016). Abiotic stress signaling and responses in plants. Cell 167, 313–324. doi: 10.1016/j.cell.2016.08.029 27716505 PMC5104190

[B58] Zhu X. Wang P. Bai Z. Herde M. Ma Y. Li N.. (2022). Calmodulin-like protein CML24 interacts with CAMTA2 and WRKY46 to regulate ALMT1-dependent Al resistance in *Arabidopsis thaliana* . New Phytol. 233, 2471–2487. doi: 10.1111/nph.17812 34665465

