# Supplementary figures and images for: Regulatory mechanism analysis of signal transduction genes during rapeseed (Brassica napus L.) germination under aluminum stress using WGCNA combination with QTL
Source: Front Plant Sci. 2025 Jan 31;16:1546572. doi: 10.3389/fpls.2025.1546572 (PMC11825321; doi:10.3389/fpls.2025.1546572)

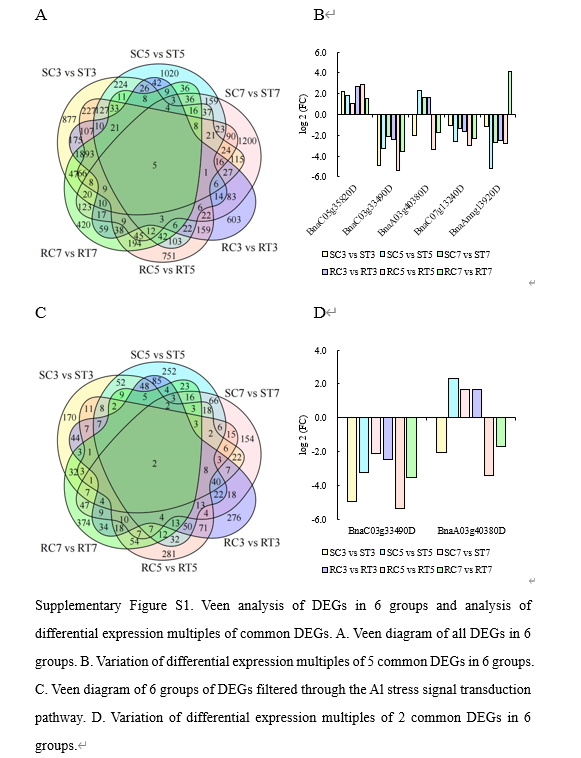

Supplement: Supplementary file 8 [file Image1.png]
